# Spleen Radiomics Signature: A Potential Biomarker for Prediction of Early and Late Recurrences of Hepatocellular Carcinoma After Resection

**DOI:** 10.3389/fonc.2021.716849

**Published:** 2021-08-13

**Authors:** Pinxiong Li, Lei Wu, Zhenhui Li, Jiao Li, Weitao Ye, Zhenwei Shi, Zeyan Xu, Chao Zhu, Huifen Ye, Zaiyi Liu, Changhong Liang

**Affiliations:** ^1^The Second School of Clinical Medicine, Southern Medical University, Guangzhou, China; ^2^Department of Radiology, Guangdong Provincial People’s Hospital, Guangdong Academy of Medical Sciences, Guangzhou, China; ^3^Department of Radiology, The Affiliated Hospital of Southwest Medical University, Luzhou, China; ^4^Department of Radiology, Yunnan Cancer Hospital, Yunnan Cancer Center, The Third Affiliated Hospital of Kunming Medical University, Kunming, China

**Keywords:** recurrence, spleen, radiomics, hepatocellular carcinoma, computed tomography

## Abstract

**Objectives:**

To explore the usefulness of spleen radiomics features based on contrast-enhanced computed tomography (CECT) in predicting early and late recurrences of hepatocellular carcinoma (HCC) patients after curative resection.

**Methods:**

This retrospective study included 237 HCC patients who underwent CECT and curative resection between January 2006 to January 2016. Radiomic features were extracted from CECT images, and then the spleen radiomics signatures and the tumor radiomics signatures were built. Cox regression analysis was performed to identify the independent risk factors of early and late recurrences. Then, multiple models were built to predict the recurrence-free survival of HCC after resection, and the incremental value of the radiomics signature to the clinicopathologic model was assessed and validated. Kaplan–Meier survival analysis was used to assess the association of the models with RFS.

**Results:**

The spleen radiomics signature was independent risk factor of early recurrence of HCC. The mixed model that integrated microvascular invasion, tumor radiomics signature and spleen radiomics signature for the prediction of early recurrence achieved the highest C-index of 0.780 (95% CI: 0.728,0.831) in the primary cohort and 0.776 (95% CI: 0.716,0.836) in the validation cohort, and presented better predictive performance than clinicopathological model and combined model. In the analysis of late recurrence, the spleen radiomics signature was the only prognostic factor associated with late recurrence of HCC.

**Conclusions:**

The identified spleen radiomics signatures are prognostic factors of both early and late recurrences of HCC patients after surgery and improve the predictive performance of model for early recurrence.

## Introduction

Hepatocellular carcinoma (HCC) is the sixth most common malignant tumor worldwide and the third leading cause of tumor-related deaths (1). Current guidelines recommend liver resection as the first option of early-stage HCC treatment for patients with preserved liver function, which can result in 5-year overall survival rates of 60%-80%. Unfortunately, tumor recurrence complicates approximately 70% of cases within five years after curative resection (2). The cut-off of two years after surgery has been adopted to grossly demarcation early recurrence (≤2 years) and late recurrence (>2 years) according to current guidelines (2, 3). Early recurrence is most likely the consequence of occult metastasis, whereas late recurrence is often the development of *de novo* tumors (4). Guidelines recommend a 3–4 months interval of follow-up in the first year after surgery for primary HCC. However, the follow-up strategies for monitoring late recurrence of HCC are still not clearly defined (2, 5). Identifying the high-risk groups of early and late recurrences of HCC can influence treatment decision and help develop personalized follow-up strategy.

Some studies (4, 6–9) investigated and compared the difference between prognostic factors of two types of recurrence, it was found that early and late recurrences were associated with different predictive factors. The predictors of early recurrence are chiefly related to primary tumor and treatment, in particular, microvascular invasion (MVI) is regarded as a critical independent risk factor of early recurrence and poor prognosis after surgery treatments of HCC (8, 10, 11). For the late recurrence, there were no clinicopathological variables that were statistically associated with recurrence. Most of the studies found that cirrhosis was the only independent prognostic factor related to late recurrence, but its prediction performance needs to be improved (4, 7, 12). Previous studies showed that noninvasive and nondestructive imaging features is closely correlated with pathological features and may rationally predict the prognosis of the tumor (13, 14). Computed tomography (CT) reflecting intrinsic tumor characters and capturing heterogeneity of tumor remain not fully developed.

Radiomics is a speedily evolving field that transforms medical imaging into high-dimensional mineable quantitative imaging features utilizing image-characterization algorithms (15). Most of the previous studies investigated the prognostic value of the radiomics based on the primary tumor (14, 16). Notably, a recent study found that splenic radiomics features based on CT can predict the prognosis of gastric cancer patients (17). As an essential immune organ of the body, the spleen can adjust inherent and acquired immunity (18). Primary tumor can promote tumor progression and metastasis by inducing inflammation and regulating immunosuppression through the spleen (19). Moreover, another study showed that spleen stiffness measurement is an independent predictor of late recurrence of HCC (8). To the best of our knowledge, no studies on the use of spleen radiomics to predict HCC recurrence have been reported to date.

This study aimed to explore the usefulness of spleen radiomics features based on contrast-enhanced computed tomography (CECT) in predicting early and late recurrences of HCC after surgery, and further evaluated if the spleen radiomics signature has incremental value to putative clinicopathological risk factors in individualized RFS estimation in HCC patients.

## Materials And Methods

### Study Population

**Figure 1:**
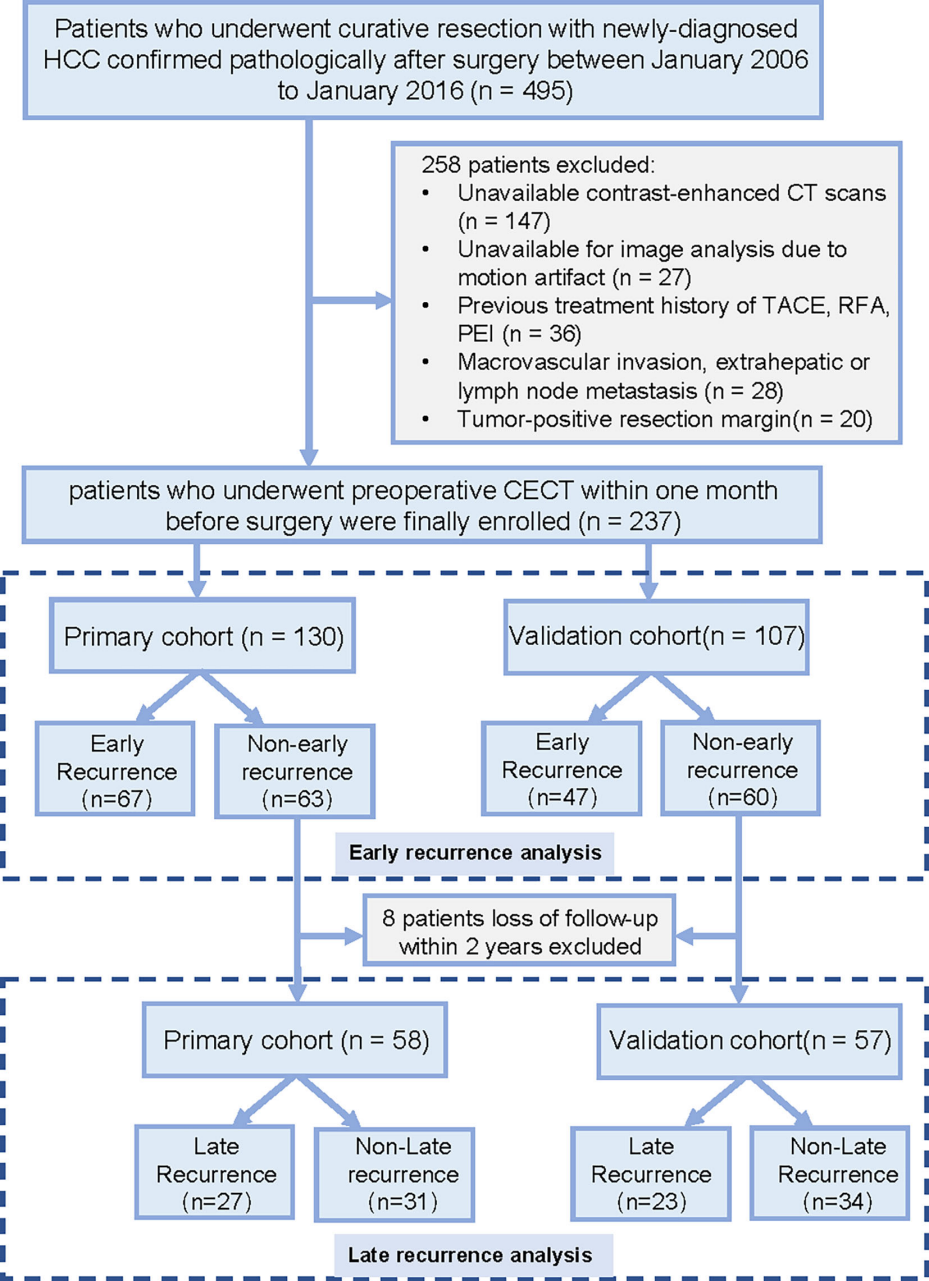
Recruitment pathway for patients in this study. HCC, hepatocellular carcinoma; CT, computed tomography; TACE, transarterial chemoembolization; RFA, radiofrequency ablation; PEI, percutaneous ethanol injection.

This retrospective cohort study was conducted in Guangdong Provincial People’s Hospital. The institutional review board approved this study and waived the requirement for written informed consent. A total of 237 patients (median age, 54.5 years; interquartile range, 45.0 –61.2 years; 203 men) were enrolled in this study. Patients who underwent surgery between January 2006 and July 2012 were assigned to the primary cohort used to construct the model, the subsequent patients from August 2012 to January 2016 were assigned to the validation cohort. The study recruitment process is shown in **Figure 1**.

Baseline clinicopathological data were collected from our picture archiving and communication system medical records. Clinical factors included age, gender, hepatitis B surface antigen (HBsAg) or hepatitis C virus antibody (HCVab) status (positive or negative) and albumin-bilirubin (ALBI) grade. The ALBI score is calculated as [-0.085 x (albumin g/l) + 0.66 x log (bilirubin umol/l)]. Based on this score, patients were stratified into 3 groups: ALBI grade 1 (a score ≤-2.60), grade 2 (>-2.60 to -1.39) and grade 3 (>-1.39) (20). Pathologic data analyzed in this study included maximum tumor diameter (mm), Edmondson-Steiner grade (I–II, III–IV), surgical margin status (positive or negative) and MVI status(absent or present) of the resected tumor. MVI was defined as the presence of tumor in the vascular space of the surrounding hepatic tissue lined by endothelium that was visible only on microscopy. Additionally, the histologic grade of liver fibrosis at the tumor periphery was reported on the basis of the Scheuer scoring system, where grades G4 indicated cirrhosis. 

### Follow-Up Surveillance

All patients underwent a clinical assessment by physical examination, laboratory exams, CECT or magnetic resonance imaging (MRI) at 3–6 months after surgery and were followed up for at least five years. We retrospectively reviewed the medical records for tumor recurrence during the follow-up. This study’s endpoint was tumor recurrence, which was diagnosed by radiologic evidence of new tumor in contrast-enhanced CT/MRI showing arterial phase hyperenhancement and wash out appearance in the portal venous phase. Recurrence-free survival (RFS) was defined as the interval from the date of surgery to the first date of tumor recurrence, metastasis on imaging (event) or the last follow-up date without recurrence (censored). We supposed that if one has an early recurrence, patients cannot have a late recurrence. The last data were censored on December 6, 2020.

### CT Imaging Protocol

CECT was carried out using three different multidetector row CT scanners. The scanning protocols are detailed in **Supplementary Material S1** and **Table S1**). Arterial phase (AP) and portal venous phase (VP) contrast-enhanced CT images with 1.25 mm reconstructed section thicknesses were selected to subsequent analysis.

### Radiomics Feature Extraction and Selection

**Figure 2:**
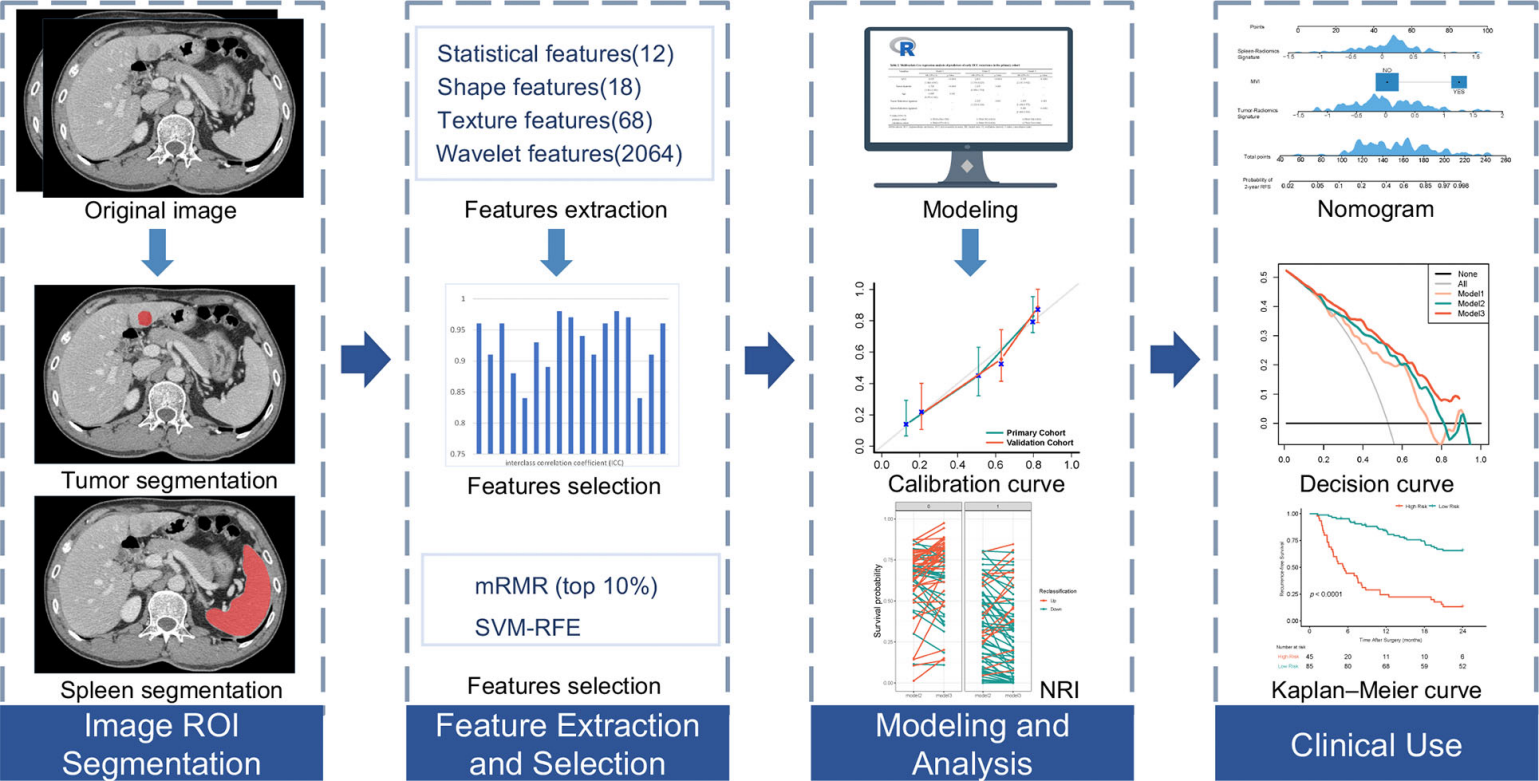
Workflow of necessary steps in current study. The regions of interest (ROIs) of tumors are segmented manually on arterial phase and portal venous phase CT section, ROI of spleens are segmented on portal venous phase. Total of 2162 radiomic features were extracted from each ROI. For features selection, features with interclass correlation coefficient >0.85 were regarded as good agreement in reproducibility and used for subsequent analysis, and the maximum relevance–minimum redundancy (mRMR) and support vector machine-recursive feature elimination (SVM-RFE) method was used to select the most critical features, the least absolute shrinkage and selection operator method was performed to construct radiomics signature. The performance of the prediction model was evaluated by the concordance index, net reclassification improvement (NRI) and calibration curve. To provide an easy-to-use assessment tool, a nomogram was built, followed by decision curve analysis and survival prediction.

The radiomics workflow is shown in **Figure 2**. For segmentation of the regions of interest (ROIs) of the primary tumor, ROIs were manually delineated along the tumor outline in the largest cross-sectional area by radiologist 1 (P. X. L, with eight years of experience in liver imaging) using ITK-SNAP software (http://www.itksnap.org). Two two-dimension (2D) region of interest (ROI-1, ROI-2) of the primary tumor were then delineated in the AP and VP, respectively, the radiologist tried to keep ROIs in the two phases as consistent as possible. For ROIs segmentation of the spleen, ROI-3 was manually delineated along the spleen outline on the slice near the splenic hilum in the VP and tried to avoid the large blood vessels of the spleen. We randomly selected 50 patients to explore the stability of each feature. Radiologist 2 (C. Z, with five years of experience in liver imaging) independently performed primary tumor and spleen segmentation to evaluate interobserver reproducibility, the reproducibility was calculated using the interclass correlation coefficient (ICC). Feature normalization was carried out using z-score normalization, feature extraction, and image preprocessing were carried out using an open-source pyradiomics package (version 2.12; https://pyradiomics.readthedocs.io/en/2.1.2/). We extracted 2162 radiomics features (12 first-order statistics, 18 shape features, 68 texture features, and 2064 wavelet decompositions features) from each ROI (ROI-1, ROI-2, ROI-3), radiomics features from the ROI-1 and ROI-2 of the primary tumor were used to construct the tumor radiomics signature. radiomics features from the ROI-3 of the spleen were used to construct the spleen radiomics signature. First, features with ICCs >0.85 were regarded as satisfactory reproducibility and high robustness and used for subsequent analysis. Next, a maximum relevance–minimum redundancy (mRMR) method was fulfilled to eliminate the redundant and irrelevant features (21), by using the mRMR method, the features were ranked according to their relevance-redundancy scores (mRMR scores), the top 10% features were selected for the following analyses. Finally, we applied the support vector machine recursive feature elimination (SVM-RFE) method to select most critical features (22). Features selection is only performed in the primary cohort.

### Radiomics Signature and Model Building

We used the least absolute shrinkage and selection operator (LASSO) Cox regression algorithm to construct the radiomics signature (radiomics score) in the primary cohort (23). Radiomics scores were set up for each patient based on a linear combination of the selected radiomics features weighted by their respective LASSO coefficients. Radiomics signature building processes were performed on both primary tumor and spleen. We respectively established two radiomics signatures based on the primary tumor and spleen for early and late recurrence analyses. Cox regression analysis was carried out to identify the independent risk factors of early recurrence and late recurrence. Only the variables with a *P*<0.05 in the univariate Cox regression analysis were enrolled in a multivariate model. For predicting RFS of recurrence, we established three models for recurrence prediction: (I) a clinicopathological model (Model 1), from clinic-pathological variables; and (II) a combined model (Model 2), from clinic-pathological variables and tumor radiomics signature; and (III) a mixed model (Model 3), from clinic-pathological variables, tumor radiomics signature and spleen radiomics signature. Subsequently, a nomogram was developed utilizing Cox regression coefficients. To ensure the independence of the primary cohort and the validation cohort, the above operations are only performed in the primary cohort. The performance of the radiomics model was validated in the validation cohort.

### Statistical Analysis

Continuous variables were reported as mean ± standard deviation and compared *via* the Student’s t-test or the Mann–Whitney U-test, categorical variables were reported as counts (percentage) and compared *via* the x^2^ test or Fisher’s exact test. Patients were stratified into high-risk and low-risk subgroups by the optimal cut-point using the survminer package of the R software. RFS probabilities were assessed by the Kaplan–Meier method, and differences between groups were compared with log-rank tests. The hazard ratios (HR) and 95% confidence interval (CI) were measured. The Harrell concordance index (C-index) was evaluated to quantify the model discrimination performance, calculation of the net reclassification improvement (NRI) was performed to evaluate the usefulness improvement added by the tumor and spleen radiomics signature. To compare the observed outcomes and the predicted RFS probabilities, calibration curves were generated. Decision curve analysis was used to assess the constructed models’ clinical usefulness (24). Two separate analyses were performed to identify the risk factors associated with early and late recurrence. For early recurrence analyses, considering all the participants enrolled. For late recurrence analyses, patients with at least two years of follow-up and without an early HCC recurrence were enrolled. All statistical and radiomics analyses were performed using the R software (R, version 3.4.2; www.r-project.org/). A two-sided *P* value less than 0.05 was considered statistically significant.

## Results

### Characteristics of the Study Cohorts

All patients’ demographic and clinicopathological characteristics are summarized in **Table 1**, clinicopathological characteristics did not differ between the primary and validation cohorts. The median duration of follow-up was 19.3 months (interquartile range, 6.4–56.0 months) for the primary cohort and 27.7 months (interquartile range, 6.7–61.2 months) for the validation cohort. 2-year and 5-year RFS were similar between the two study cohorts (*P* = 0.243,0.502, respectively). Recurrences occurred in 164 patients (early recurrence, 114; late recurrence, 50) of the 237 patients, with assessed 2-, and 5-year cumulative RFS rates being 51.9%, and 30.9%, respectively. The median follow-up of 115 patients at least two years of follow-up without an HCC early recurrence was 61.1 months (interquartile range, 36.5–84.9 months).

**Table 1:** Baseline characteristics of the HCC patients in the primary and validation cohorts.

Characteristics	Primary cohort (n = 130)	Validation cohort (n = 107)	*p* Value
Age (year)*	53.95 ± 13.23	52.89 ± 12.58	0.532
Sex			0.381
Female	21 (16.2%)	13(12.1%)	
Male	109 (83.8%)	94 (87.9%)	
Tumor diameter (mm)*	52.90 ± 31.76	52.22 ± 42.65	0.889
MVI			0.558
Absent	89 (68.5%)	77 (72.0%)	
Present	41 (31.5%)	30 (28.0%)	
Edmondson grade			0.603
I–II	42 (32.3%)	38 (35.5%)	
III–IV	88 (67.7%)	69 (64.5%)	
Cirrhosis			0.689
Absent	61 (46.9%)	53 (49.5%)	
Present	69 (53.1%)	54 (50.5%)	
HBsAg or HCVab status			0.058
Negative	18 (13.8%)	25 (23.4%)	
Positive	112 (86.2%)	82 (76.6%)	
ALBI grade			0.269
1	34 (26.2%)	35 (32.7%)	
2 or 3	96 (73.8%)	72 (67.3%)	

HCC, hepatocellular carcinoma; MVI, microvascular invasion; ALBI, albumin-bilirubin.

Except where indicated, data are numbers of patients, with percentages in parentheses. *Continuous variables are expressed as mean ( ± standard deviation).

### Clinicopathologic Prognostic Factors

Univariate analyses were performed on all the clinicopathologic variables in the primary cohort (including age, gender, ALBI grade, tumor diameter, MVI, Edmondson-Steiner grade, HBsAg or HCVab status, cirrhosis). Only the variables with a *P*<0.05 in the univariate Cox regression analysis were enrolled in a multivariate model. For early recurrence, MVI, tumor diameter and age were found significantly associated with early recurrence in univariate analysis (*P*<0.01, shown in **Table S2**). Then, we built a clinicopathologic model (called Model 1) using MVI and tumor diameter with backward stepwise selection. The identified independent risk factors of early recurrence were MVI (HR: 3.037, 95% CI: 1.860, 4.961, *P <*0.0001) and tumor diameter (HR: 1.723, 95% CI: 1.361, 2.181, *P <*0.0001) (**Table 2**). For late recurrence, no clinicopathological variables were significantly associated with recurrence (**Table S3**).

**Table 2:** Multivariate Cox regression analysis of early recurrence of HCC in the primary cohort.

Variables	Model 1		Model 2		Model 3	
	HR (95% CI)	*p* Value	HR (95% CI)	*p* Value	HR (95% CI)	*p* Value
MVI	3.037 (1.860-4.961)	<0.0001	2.815 (1.714-4.625)	<0.0001	3.575 (2.151-5.942)	<0.0001
Tumor diameter	1.723 (1.361-2.181)	<0.0001	1.227 (0.858-1.754)	0.263	NA	NA
Age	0.987 (0.971-1.001)	0.142	NA	NA	NA	NA
Tumor-radiomics signature	NA	NA	2.237 (1.213-4.126)	0.010	1.997 (1.058-3.772)	0.033
Spleen-radiomics signature	NA	NA	NA	NA	3.285 (1.960-5.507)	<0.0001
C-index (95% CI)						
Primary cohort	0.735 (0.676-0.795)		0.759 (0.702-0.815)		0.780 (0.728-0.831)	
Validation cohort	0.744 (0.677-0.811)		0.764 (0.703-0.824)		0.776 (0.716-0.836)	

HCC, hepatocellular carcinoma; MVI, microvascular invasion; HR, hazard ratio; CI, confidence interval; C-index, concordance index; NA, not applicable.

### Radiomics Feature Selection and Signature Construction

**Figure 3:**
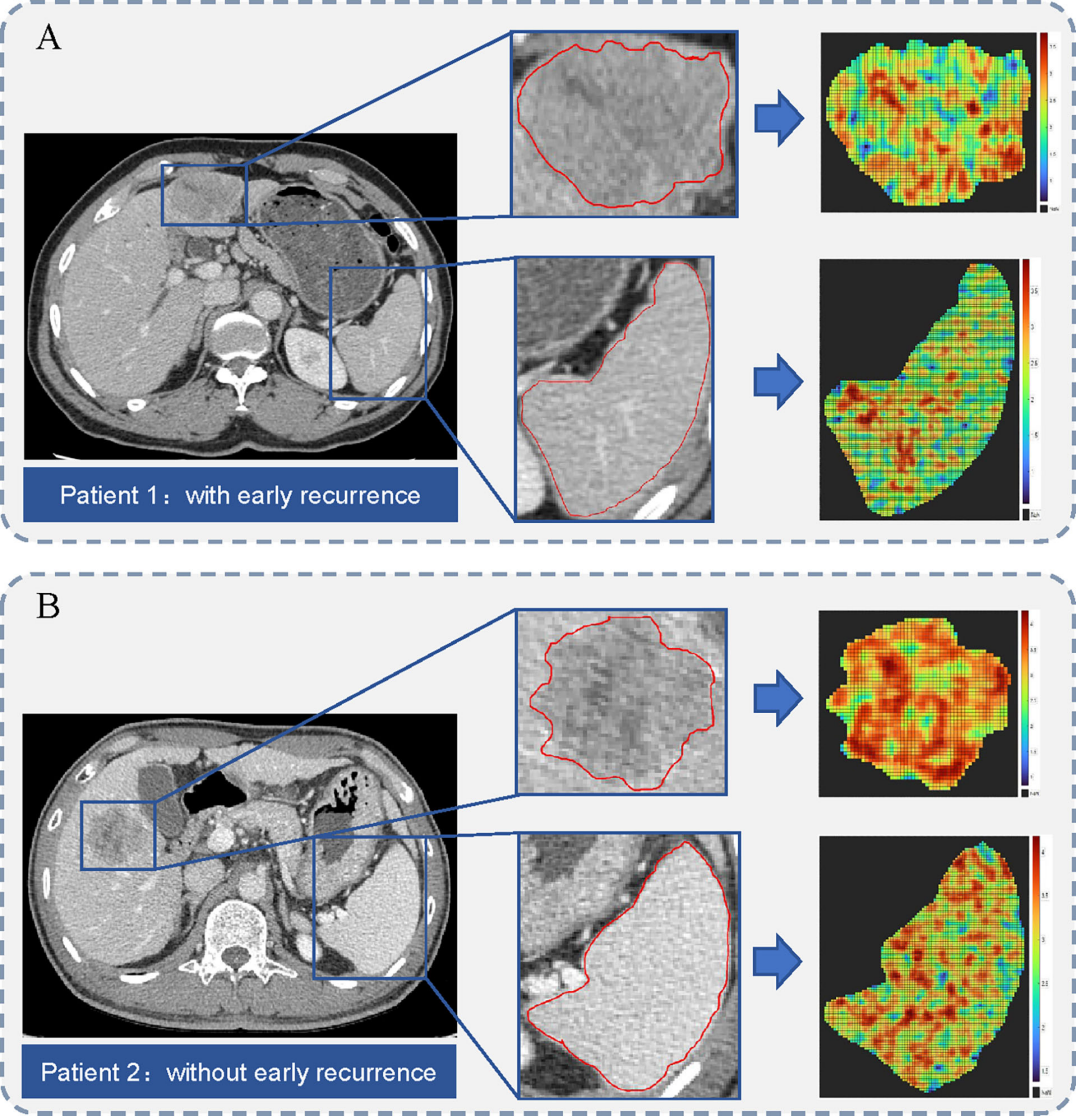
Illustration of region of interest (ROI) segmentation for two representative cases. The middle column shows the tumor and spleen outlines on the original CT image. The right column shows the heat maps of radiomics score on tumor and spleen images. **(A)** A 68-year-old man with microvascular invasion (MVI) and a 49-mm liver mass. Hepatic metastasis occurred 12.4 months after surgery. **(B)** A 48-year-old man with MVI and a 40-mm liver mass. He remained recurrence-free during 74.3 months of follow-up period after surgery.

Among the 4324 and 2162 extracted radiomics features from the primary tumor and the spleen, respectively. A total of 3220 features with high stability from the primary tumor, and 1992 features with high stability from the spleen were preliminarily selected. The most critical features were selected by applying the SVM-RFE and mRMR method. Finally, four radiomics signature (two tumor radiomics signature and two spleen radiomics signature) was constructed using the LASSO regression algorithm for the estimation of early and late recurrence, respectively. The formula for radiomics signature and the coefficients of the corresponding feature are described in **Supplementary Material S2**. The representative cases of ROI segmentation and radiomics score heat maps are shown in **Figure 3**.

### Radiomics Analysis and Model Comparison

**Figure 4:**
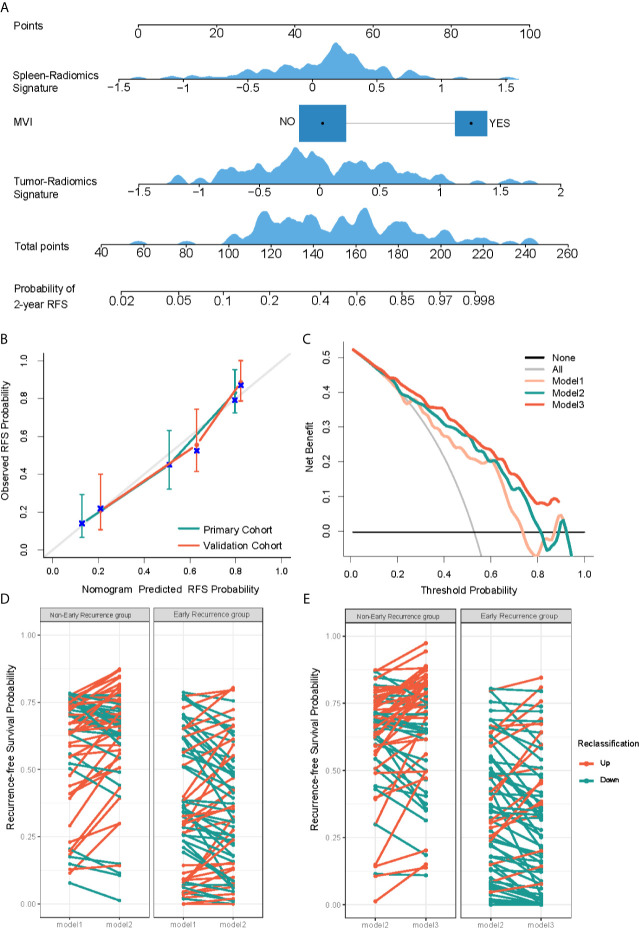
Development and performance evaluation of nomogram for recurrence-free survival (RFS) evaluation of HCC early recurrence. **(A)** A radiomics nomogram was developed in the primary cohort, with spleen-radiomics signature, tumor-Radiomics Signature and microvascular invasion (MVI) incorporated. **(B)** Calibration curves to show the calibration of the radiomics nomogram in terms of the agreement between the predicted and the observed 2-year RFS in both primary and validation cohorts. **(C)** Decision curve analysis of clinical usefulness assessment of Model 3 in the primary cohort, the y-axis represents the net benefit, and the x-axis represents the threshold probability. Model 3 achieves more net benefit across the majority of the range of threshold probabilities compared with Model 1, Model 2, treat-all strategy (gray line), and treat-none strategy (horizontal black line). **(D, E)** Net reclassification improvement (NRI) analysis of models in the primary cohort. Potential incremental value of Model 2 relative to Model 1 **(D)** and Model 3 to Model 2 **(E)** were assessed by NRI.

**Figure 5:**
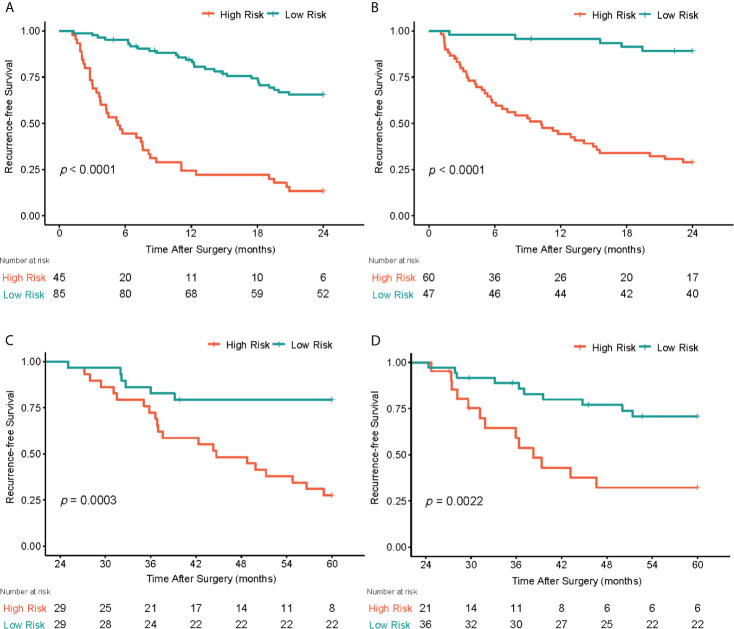
Graphs show recurrence-free survival (RFS) of patients. In early postoperative periods (<2 years), patients were stratified into low-risk and high-risk subgroups by the optimal cut-point according to the radiomics nomogram, the survival curves of the low-risk and high-risk groups were significantly different in the primary **(A)** and validation **(B)** cohorts. In late postoperative periods (>2 years), patients were successfully stratified into low-risk and high-risk groups according to the spleen radiomics signature in the primary **(C)** and validation **(D)** cohorts.

For the prediction of RFS in early recurrence, three prediction models were constructed. The C-index of Model 1 were 0.735 (95% CI: 0.676,0.795) and 0.744 (95% CI: 0.677,0.811) in the primary and validation cohorts, respectively. After integrating tumor radiomics signature into Model 1, Model 2 achieved higher prognostic performance with a C-index of 0.759 (95% CI: 0.702,0.815) and 0.764 (95% CI: 0.703,0.824) in the primary and validation cohorts. Similarly, by incorporating spleen radiomics signature into Model 2, Model 3 yielded the highest C-index of 0.780 (95% CI: 0.728,0.831) and 0.776 (95% CI: 0.716,0.836) in the primary and validation cohorts (**Table 2**). The NRI was calculated to evaluate the usefulness improvement of model added by the tumor and spleen radiomics signature, Model 2 show significantly improved classification performance than Model 1 in the primary (NRI,0.296, 95% CI: -0.242, 0.681) and validation (NRI,0.053, 95% CI: -0.384, 0.323) cohorts. Similarly, Model 3 showed better classification performance than Model 2 in the primary (NRI,0.484,95%CI:0.076,0.910) and validation (NRI,0.359,95%CI: -5.059,0.716) cohorts. The reclassification of the prediction models is shown in **Figures 4D, E** and **Figure S1**. Finally, a nomogram based on Model 3 was built (shown in **Figure 4A**). The nomogram calibration curve for the probability of 2-year RFS in the primary and validation cohorts demonstrated good agreement between prediction and observation (**Figure 4B**). A decision curve analysis showed that Model 3 achieves more net benefit across the majority of the range of threshold probabilities compared with Model 1, Model 2, treat-all strategy, and treat-none strategy in the primary cohort (**Figure 4C**). According to the optimal cutoff value of the Model 3, all 237 patients were successfully stratified into low-risk and high-risk subgroups by the optimal cut-point, the survival curves of the low-risk and high-risk groups were significantly different in the primary (log-rank test, *P*<0.0001; **Figure 5A**) and validation cohorts (log-rank test, *P*<0.0001; **Figure 5B**).

For the prediction of RFS in late recurrence. The spleen radiomics signature was the only prognostic factor associated with RFS in late recurrence analysis, it achieved a C-index of 0.661 (95% CI: 0.562,0.759) in the primary cohort and 0.688 (95% CI: 0.576,0.800) in the validation cohort. Of the 115 patients enrolled in late recurrence analyses, the patients were stratified into low- and high-risk group based on the optimal cut-off values of spleen radiomics scores defined from the primary cohort, the low-risk group showed higher RFS in the primary cohort (log-rank test, *P*<0.0003; **Figure 5C**) and the validation cohort (log-rank test, *P*=0.002; **Figure 5D**). However, our analysis showed that the tumor radiomics signature did not show statistically significant association with late recurrence.

## Discussion

In the present study, we demonstrated that the splenic radiomics signatures were potential prognostic factors of both early and late recurrences of HCC. The model that incorporated the spleen radiomics signature exhibited better prognostic performance in both study cohorts (C-index=0.776,0.780, respectively) for prediction of early recurrence. For late recurrence analyses, we found that the spleen radiomics signature was the only prognostic factor. The constructed nomogram and the spleen radiomics signature could respectively categorize HCC patients into two recurrence risk subgroups in early and late postoperative periods.

The present study found that MVI and primary tumor diameter were independent risk factors for the early recurrence of HCC, and then a clinicopathological model based on those two factors was built. For the late recurrence, there were no clinicopathological variables that were statistically associated with recurrence in present study. Early recurrence, which is likely the consequence of occult metastasis from the original tumor, can be predicted by intrinsic tumor characteristics. Whereas, late recurrence is often attributed to the development of a *de novo* tumor which is different from the original tumor (4, 10). Our results confirmed findings of other studies (6, 10, 11) that MVI and tumor size are independent factor for early recurrence. MVI is the single parameter enrolled by multiple models (25–27). For risk factors associated with late recurrence, the presence of cirrhosis has already been investigated and was related to a higher risk of late recurrence (4, 7, 28). Nevertheless, cirrhosis was not statistically associated with HCC late recurrences in present study.

Radiomics features can quantify the tumor heterogeneity by using image-characterization algorithms (15). Several studies (14, 16, 29) have evaluated the prognostic value of radiomics derived from the primary tumor of HCC patients and demonstrated the incremental value of the radiomics signature to the clinicopathological characteristics, but most of those only investigated the efficiency of radiomics in predicting the early recurrence of HCC. A recent study demonstrated that the CT-based radiomics models showed superior prognostic performance (C-index ≥0.77) than other models in predicting the HCC recurrence (14). In an earlier radiomics study, the combined clinicopathologic-radiomics model based on gadoxetic acid–enhanced MRI achieved a C-index of 0.716 in predicting DFS of early recurrence of HCC (7). The mixed model that incorporated the spleen radiomics signature achieved the highest prediction performance (C-index=0.776,0.780; in two cohorts) for the prediction of early recurrence in the present study. For late recurrence analyses, only a few studies have investigated the potential role of radiomics in predicting HCC late recurrence, and with a limited value (7). Notably, our study demonstrated that the spleen radiomics signature was the only prognostic factor of late recurrence of HCC.

Most of the previous radiomics studies only assessed the prognostic value of radiomics features extracted from the primary tumor or peritumoral (14, 29). To the best of our knowledge, this study is the first to report the use of splenetic radiomics for the assessment of the prognostic value of HCC recurrence, our results indicated that the spleen radiomics signatures were effective predictors of early and late recurrences. The secondary change of splenic microenvironment and architecture heterogeneity is still unclear in tumor patients (18, 30). The primary tumor of HCC patients can promote tumor progression and metastasis by inducing inflammation and regulating immunosuppression through the spleen (18, 19). Wang et al. demonstrated that the splenic radiomics model was an independent risk factor for the survival of patients with gastric cancer (17), and spleen density can predict the overall survival of patients with gastric cancer (31). All of the above suggests that the spleen has a close biological relationship with primary tumors’ development. Also, it is widely accepted that the presence and the degree of portal hypertension were significantly correlated to HCC occurrence (32). Late recurrence is possibly associated with the evolution of the potential chronic liver disease. A related study showed that the spleen stiffness measurement, which indirectly reflects portal hypertension, was an independent predictor of the late recurrence of HCC (8). And the so-called “liver-spleen axis” is arousing increasing attention in liver disease (33). Previous study has verified that CT-based splenic radiomics can non-invasively predict portal venous pressure (34, 35), which may explain why the splenic signature was associated with HCC patient prognosis. The advantage of using splenic radiomics signature is not affected by primary tumor size and can be easily obtained before operation with simple processing.

To provide clinicians with an easy-to-use assessment tool, a nomogram based on Model 3 was built in our study. our nomogram can enable clinicians design a tailored surveillance program mainly for those patients with a high risk of recurrence. Although the usefulness of the proposed nomogram lacked independent external validation, the decision curve analysis proved that the mixed model was superior to both the combined radiomics model and the clinicopathologic model across the majority of the reasonable range threshold probabilities.

There are limitations to our study. First, our analysis was performed in China. Most patients had a background of hepatitis B virus–related HCC, which is not the main etiological factor of HCC occurrence in other countries or regions. Second, radiomics features were extracted from the largest cross-sectional area in place of the whole tissue, the three-dimensional ROI segmentation may provide more information about tissues, but 2D features are easier to obtain. Finally, due to the small sample size and the lack of independent external validation in multicenter may affect the robustness and generalizability of our outcomes. A larger cohort population of the prospective study is needed to validate the result in the future.

In conclusion, the identified spleen radiomics signatures were significant prognostic factors of both early and late recurrences of HCC patients after curative resection. The spleen radiomics signature’s incremental value for RFS prediction of early recurrence was demonstrated, the proposed nomogram can be used as an easy-to-use and visual tool to develop personalized follow-up strategies and guide clinical decision-making.

## Data Availability Statement

The raw data supporting the conclusions of this article will be made available by the authors, without undue reservation.

## Ethics Statement

The studies involving human participants were reviewed and approved by the Institutional Review Board of Guangdong Provincial People’s hospital. Written informed consent for participation was not required for this study in accordance with the national legislation and the institutional requirements.

## Author Contributions

PL and LW designed the study. PX, LW, and ZHL wrote the draft report. CL and ZYL initiated and coordinated the study. PL, JL, CZ, and HY collected data and labeled images. PL, LW,WY, ZS, and ZX analyzed images. LW did the statistical analysis. All authors discussed the early version of the report and provided comments and suggestions for change. All authors contributed to the article and approved the submitted version.

## Funding

This work was supported by the National Key Research and Development Program of China [2017YFC1309100], the National Science Fund for Distinguished Young Scholars [81925023], the National Natural Science Foundation of China [82071892] and the National Science Foundation for Young Scientists of China [81701662,82001986], the Project funded by China Postdoctoral Science Foundation [2020M682643].

## Conflict of Interest

The authors declare that the research was conducted in the absence of any commercial or financial relationships that could be construed as a potential conflict of interest.

## Publisher’s Note

All claims expressed in this article are solely those of the authors and do not necessarily represent those of their affiliated organizations, or those of the publisher, the editors and the reviewers. Any product that may be evaluated in this article, or claim that may be made by its manufacturer, is not guaranteed or endorsed by the publisher.

## Abbreviations

HCC, Hepatocellular carcinoma; RFS, Recurrence-free survival; MVI, Microvascular invasion; CT, Computed tomography; CECT, Contrast-enhanced computed tomography; MRI, Magnetic resonance imaging; AP, Arterial phase; VP, Portal venous phase; ROI, Region of interest; 2D, Two-dimension; ICC, Interclass correlation coefficient; mRMR, Maximum relevance–minimum redundancy; SVM-RFE, Support vector machine recursive feature elimination; LASSO, Least absolute shrinkage and selection operator; HR, Hazard ratio; CI, Confidence interval; C-index, Concordance index; NRI, Net reclassification improvement.
