# Supplementary material for: Spleen Radiomics Signature: A Potential Biomarker for Prediction of Early and Late Recurrences of Hepatocellular Carcinoma After Resection
Source: Front Oncol. 2021 Aug 13;11:716849. doi: 10.3389/fonc.2021.716849 (PMC8414994; doi:10.3389/fonc.2021.716849)
Supplement: Supplementary file 1 [file DataSheet_1.docx]

**Supplementary Material**

**S1. Supplementary Methods**

**CT protocols of the three scanners**

Contrast-enhanced CT was carried out using one of the following multidetector row CT scanners: 64-slice LightSpeed VCT (GE Medical systems, Milwaukee, WI); Lightspeed Ultra 8 (GE Healthcare, Hino, Japan), or Brilliance iCT 256 (Philips Healthcare, Cleveland OH, USA). The scanning protocols used at the three scanners are detailed in table S1. Following a routine-unenhanced CT, A volume of 1.5 mL/kg body weight of contrast material (Ultravist 370, Bayer Schering Pharma, Berlin, Germany) was injected through an intravenous antecubital cannula with a rate of 3.0 to 3.5 mL/s via a pump injector (Ulrich CT Plus 150, Ulrich Medical, Ulm, Germany). Arterial phase and portal venous phase contrast-enhanced CT images were performed at 25–30 s and 60–70 s, respectively, and with 1.25 mm reconstructed section thicknesses of the arterial phase and portal venous phase.

**S2. Supplementary** **Results**

**Radiomic Signature (Score) Calculation Formula**

Four radiomics signature (two tumor radiomics signature and two spleen radiomics signature) was constructed using the least absolute shrinkage and selection operator regression algorithm for the estimation of early and late recurrence, respectively.

For early recurrence analyses：

Tumor Radiomics Signature= 0.425×AP_rbio5.5.LH_glszm_SizeZoneNonUniformity

+ 0.206× VP_rbio5.5.LH_glszm_SmallAreaEmphasis

—0.230×AP_db4.LL_gldm_SmallDependenceEmphasis

—0.200×AP_coif3.HH_glcm_Imc2

Spleen Radiomics Signature= —0.300×VP_glrlm_RunVariance

+ 0.354×VP_rbio5.5.LH_firstorder_Minimum

—0.158×VP_bior2.8.HL_gldm_GrayLevelVariance

—0.105×VP_rbio5.5.HH_glrlm_ShortRunEmphasis

For late recurrence analyses：

Tumor Radiomics Signature= —0.544×VP_coif3.HH_glszm_GrayLevelVariance

+0.393×VP_db4.LL_glszm_GrayLevelNonUniformity

+0.233×AP_db4.LL_firstorder_Minimum

Spleen Radiomic Signature= —0.660×VP_db4.HL_glszm_ZonePercentage

—0.263×VP_original_shape_Elongation

+0.140×VP_db4.HL_glszm_SizeZoneNonUniformityNormalized

**TABLE** **S1.** Scanning parameters for each scanner

| **Parameter** | **Brilliance iCT 256** |  | **Lightspeed Ultra 8** |  | **64-Slice LightSpeed VCT** |
| --- | --- | --- | --- | --- | --- |
| Tube voltage (kVp)  Tube current (mA)  Detector collimation (mm)  Field of view (mm)  Pixel size  Rotation time (s)  Slice interval (mm)  Slice thickness (mm)  Reconstructed section thicknesses (mm) | 120  Auto  128 × 0.625  300–400  512 × 512  0.5  5  5  1.25 |  | 120  240  8 × 0.625  300–400  512 × 512  0.6  5  5  1.25 |  | 120  Auto  64 × 0.625  300–400  512 × 512  0.4  5  5  1.25 |

|  | **Primary cohort（n=130）** | | | **Validation cohort（n=107）** | | |
| --- | --- | --- | --- | --- | --- | --- |
| **Characteristics** | **Early Recurrence（n=67）** | **Non-Early Recurrence（n=63）** | ***p* Value** | **Early Recurrence（n=47）** | **Non-Early Recurrence（n=60）** | ***p* Value** |
| Age (year)* | 51.13 ± 13.26 | 56.94 ± 12.64 | 0.012^†^ | 52.53 ± 12.30 | 53.85 ± 11.05 | 0.561 |
| Sex |  |  | 0.695 |  |  | 0.672 |
| Female | 10 (14.9%) | 11 (17.5%) |  | 5 (10.6%) | 8 (13.3%) |  |
| Male | 57 (85.1%) | 52 (82.5%) |  | 42 (89.4 %) | 52 (86.7%) |  |
| Tumor diameter (mm)* | 64.31 ± 34.02 | 40.76 ± 24.00 | <.001^†^ | 62.45 ± 34.35 | 39.13 ± 22.03 | <.001^†^ |
| MVI |  |  | <.001^†^ |  |  | <.001^†^ |
| Absent | 36 (53.7%) | 53 (84.1%) |  | 23 (48.9%) | 54 (90.0%) |  |
| Present | 31 (46.3%) | 10 (15.9%) |  | 24 (51.1%) | 6 (10.0%) |  |
| Edmondson grade |  |  | 0.171 |  |  | 0.090 |
| I–II | 18 (26.9%) | 24 (38.1%) |  | 17 (36.2%) | 21 (35.0%) |  |
| III–IV | 49 (73.1%) | 39 (61.9%) |  | 30 (63.8%) | 39 (65.0%) |  |
| Cirrhosis |  |  | 0.368 |  |  | 0.503 |
| Absent | 34 (50.7%) | 27 (42.9%) |  | 25 (53.2%) | 28 (46.7%) |  |
| Present | 33 (49.3%) | 36 (57.1%) |  | 22 (46.8%) | 32 (53.3%) |  |
| HBsAg or HCVab status |  |  | 0.247 |  |  | 0.639 |
| Negative | 7(10.4%) | 11(17.5%) |  | 12(25.5%) | 13(21.7%) |  |
| Positive | 60(89.6%) | 52(82.5%) |  | 35(74.5%) | 47(78.3%) |  |
| ALBI grade |  |  | 0.543 |  |  | 0.568 |
| 1 | 16 (23.9%) | 18 (28.6%) |  | 14 (29.8%) | 21 (35.0%) |  |
| 2 or 3 | 51 (76.1%) | 45 (71.4%) |  | 33 (70.2%) | 39 (65.0%) |  |

**TABLE** **S2.** Clinical characteristics between early recurrence and non-early recurrence groups in the primary and validation cohorts

*MVI*, microvascular invasion; *ALBI*, albumin-bilirubin.

Except where indicated, data are numbers of patients, with percentages in parentheses. *P* value is derived from the univariable association analyses between each of the clinicopathological variables and early recurrence.

* Continuous variables are expressed as mean (±standard deviation).

^†^ *P* value < .05.

**TABLE S3.** Clinical characteristics between late recurrence and non-late recurrence groups in the primary and validation cohorts

|  | **Primary cohort（n=58）** | | | **Validation cohort（n=57）** | | |
| --- | --- | --- | --- | --- | --- | --- |
| **Characteristics** | **Late Recurrence（n=27）** | **Non-Late Recurrence（n=31）** | ***p* Value** | **Late Recurrence（n=23）** | **Non-Late Recurrence（n=34）** | ***p* Value** |
| Age (year)* | 56.15 ± 12.23 | 57.58 ± 13.42 | 0.674 | 53.57 ± 11.05 | 54.24 ± 10.45 | 0.817 |
| Sex |  |  | > 0.99 |  |  | > 0.99 |
| Female | 5 (18.5%) | 5 (16.1%) |  | 3 (13.0%) | 5(14.7%) |  |
| Male | 22 (81.5%) | 26 (83.9%) |  | 20 (87.0 %) | 29 (85.3%) |  |
| Tumor diameter (mm)* | 42.26 ± 24.92 | 40.84 ± 25.03 | 0.830 | 36.35 ± 19.39 | 41.62 ± 24.49 | 0.391 |
| MVI |  |  | 0.311 |  |  | >0.99 |
| Absent | 24 (88.9%) | 24 (77.4%) |  | 21 (91.3%) | 31 (91.2%) |  |
| Present | 3(11.1%) | 7 (22.6%) |  | 2 (8.7%) | 3(8.8%) |  |
| Edmondson grade |  |  | 0.340 |  |  | 0.409 |
| I–II | 12 (44.4%) | 10 (32.3%) |  | 7 (30.4%) | 14 (41.2%) |  |
| III–IV | 15 (55.6%) | 21 (67.7%) |  | 16 (69.6%) | 20 (58.8%) |  |
| Cirrhosis |  |  | 0.079 |  |  | 0.413 |
| Absent | 7 (25.9%) | 15(48.4%) |  | 12 (52.2%) | 14 (41.2%) |  |
| Present | 20(74.1%) | 16 (51.6%) |  | 11 (47.8%) | 20 (58.8%) |  |
| HBsAg or HCVab status |  |  | 0.481 |  |  | 0.744 |
| Negative | 3(11.1%) | 6(19.4%) |  | 4(17.4%) | 8(23.5%) |  |
| Positive | 24(88.9%) | 25(80.6%) |  | 19(82.6%) | 26(76.5%) |  |
| ALBI grade |  |  | 0.268 |  |  | 0.703 |
| 1 | 6 (22.2%) | 11 (35.5%) |  | 7 (30.4%) | 12 (33.3%) |  |
| 2 or 3 | 21 (77.8%) | 20(64.5%) |  | 16 (69.6%) | 22 (66.7%) |  |

*MVI*, microvascular invasion; *ALBI*, albumin-bilirubin.

Except where indicated, data are numbers of patients, with percentages in parentheses. *P* value is derived from the univariable association analyses between each of the clinicopathological variables and late recurrence.

* Continuous variables are expressed as mean (±standard deviation).

A B


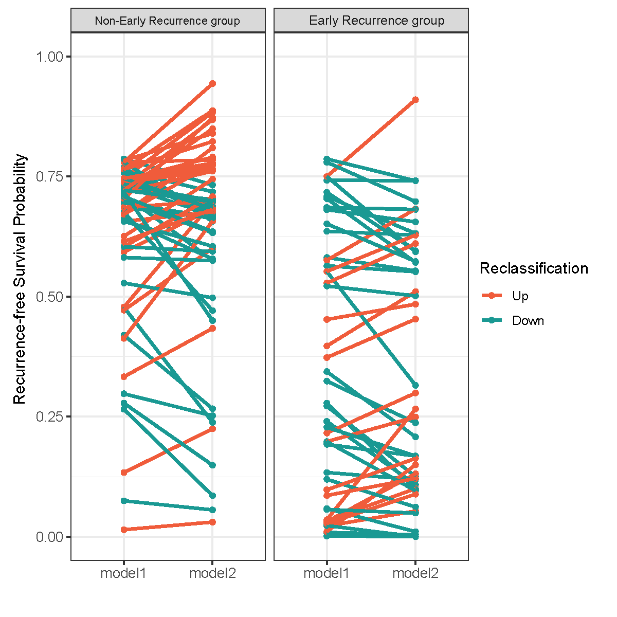

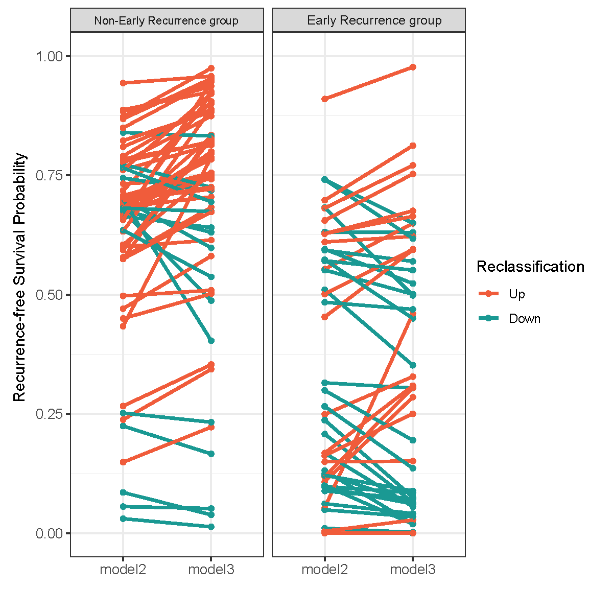


**FIGURE S1**. Net reclassification improvement (NRI) analysis of models in the validation cohort. Potential incremental value of Model 2 relative to Model 1(**A**) and Model 3 to Model 2(**B**) were assessed by NRI.
